# Amelioration of diabetic nephropathy in db/db mice treated with tibetan medicine formula Siwei Jianghuang Decoction Powder extract

**DOI:** 10.1038/s41598-018-35148-2

**Published:** 2018-11-12

**Authors:** Xianrong Lai, Dong Tong, Xiaopeng Ai, Jiasi Wu, Yu Luo, Fang Zuo, Zhicheng Wei, Yanqiao Li, Wanyi Huang, Wenqian Wang, Qing Jiang, Xianli Meng, Yong Zeng, Ping Wang

**Affiliations:** 1 School of Pharmacy, Chengdu University of Traditional Chinese Medicine, Chengdu, 611137 China; 2 School of Ethnic Medicine, Chengdu University of Traditional Chinese Medicine, Chengdu, 611137 China

## Abstract

Siwei Jianghuang Decoction Powder (SWJH) documented originally in *the Four Medical Tantras-Blue Glaze* exhibited beneficial effects on diabetic nephropathy (DN) via combined synergistically action of multiple formula components including Curcumae longae Rhizoma, Berberidis dictyophyllae Cortex, Phyllanthi Fructus and Tribuli Fructus. This study investigated the effects of SWJH on DN in db/db mice and possible underlying mechanisms. The ten weeks old db/db mice treated with SWJH by intra-gastric administration once a day for 8 weeks. After 8 weeks, body weight, water and food intake of mice were recorded. The level of fasting blood glucose (FBG) was measured. Serum creatinine (Scr), blood urea nitrogen (BUN), urine microalbumin (UMAlb), serum uric acid (UA) and urinary albumin excretion (UAE) were detected. An enzyme-linked immunosorbent assay was performed to test serum vascular endothelial growth factor (VEGF) and transforming growth factor-β1 (TGF-β1). Real-time PCR and Western blot analysis were used to test mRNA and protein expression of hypoxia inducible factor-1α (HIF-1α), VEGF and TGF-β1 in kidney tissue. SWJH treatment significantly reduced the levels of FBG, Scr, BUN, UMAlb, UA and UAE and retarded renal fibrosis. SWJH treatment further significantly reduced serum TGF-β1 level and downregulated the expression of HIF-1α, VEGF and TGF-β1 at both mRNA and protein levels. Principal component analysis and partial least squares regression and hierarchical cluster analysis demonstrated that SWJH treatment significantly ameliorated renal damage in DN mice. These consequences suggested that SWJH formulations were effective in the treatment of DN through regulating the HIF-1α, VEGF and TGF-β1 overexpression.

## Introduction

The international diabetes federation (IDF) estimated that 642 million adults worldwide suffered from diabetes mellitus (DM) in 2040. The complications of DM divided into microvascular damages and macrovascular damages. Microvascular endothelial damage is the key link of diabetic nephropathy (DN)^1^. DN is one of the main reasons the progression of DM towards end-stage renal disease (ESRD) and the most common cause of kidney failure and it is a major threat to human health^2,3^. It is believed that persistent hyperglycemia plays a positive role in the pathogenesis of DN, but simply controlling blood glucose doesn’t stop the progression of DN symptoms. Because many factors are involved in the occurrence and development of DN, including the increased vascular permeability and other vascular endothelial dysfunction, which play a major role in the pathogenesis of DN^4^. Despite the increased research effort and active clinical trials concerning a variety of drugs, the progression of DN towards ESRD remains unclear^5,6^. Therefore, it is a critical need to develop novel kidney protection intervention methods and drugs for DN treatment.

The major pathological changes of DN refer to the glomerulus sclerosis caused by diabetic microvascular disease, thickness of the glomerular basement membrane (GBM) and occurring endothelial damage^7^. These can cause glomerular filtration barrier (GFB) dysfunction and then contribute to the occurrence and development of micro-albuminuria, numerous research demonstrate that transforming growth factor-β1 (TGF-β1) has been identified as a key regulator of fibrosis in DN or renal disease^8–11^. A lot of evidence from animal models of diabetes and human patients suggested that persistent hyperglycemia increases the expression of renal TGF-β1^12^. In addition, vascular endothelial growth factor (VEGF) is the main factor that linked to DN, the clinical study shown that, at the early stage of DN diabetic urine VEGF level is raised, and the level of urinary VEGF and DN lesions in diabetic patients have clinical relevance, urinary VEGF high expression levels leading to pathological GBM structure change, and produces a large of albumin^13,14^. Hypoxia inducible factor-1α (HIF-1α) is a transcription factor induced by hypoxia reaction, which can activate expression of many hypoxia responsive genes and involve in the development of DN, it is also closely related to renal function decline and proteinuria appear. In addition, in the occurrence and development of DN, HIF-1α has a regulate effect on VEGF, TGF-β1, the key factors^15,16^. Thus, an array of drug candidates, which defeat DN with few side effects via the modulation of HIF-1α/VEGF/TGF-β1 signaling pathways, need to be further developed.

**Figure 1:**
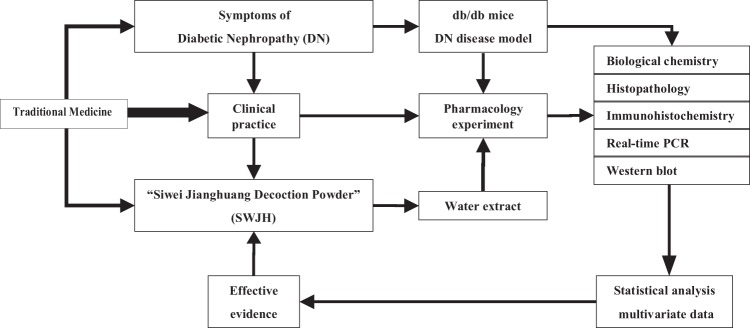
Technical roadmap of amelioration of DN in db/db mice treated with tibetan formula Siwei Jianghuang extract.

The practice of traditional Chinese medicine (TCM) is largely guided by the cumulative empirical experience of its practitioners. TCM are frequently used for treatment of kidney diseases in China and many other Asian countries^17–21^. “Siwei Jianghuang Decoction Powder” (SWJH, Tibetan herbal medicine prescription containing Curcumae Longae Rhizoma, Berberidis dictyophyllae Cortex, Phyllanthi Fructus and Tribuli Fructus, herbal dosage ratio was 1:2:1:2), used to correct imbalance condition of symptoms accompanied by DN occurrence and development. Curcumae Longae Rhizoma can reduce the kidney hypertrophy index of DN induced by streptozocin(STZ) and reduce the level of urinary albumin excretion rate (UAER), its protective effect on DN mainly by reduced the overexpression of TGF-β1 and prevented renal fibrosis in DN^22,23^. The results of the preliminary pharmacological test shown that Berberidis dictyophyllae Cortex shown a significant effect on reducing hypoglycemic and improving microangiopathy pathology in DN, and inhibited the expression of cytokines related to diabetic microangiopathy such as HIF-1α and VEGF^24^. Phyllanthi Fructus and Tribuli Fructus play a critical role in lowering blood glucose^25,26^. The preliminary research in our research team shown that SWJH provided a evidence on renal injury of DN in rats induced by STZ intraperitoneally with a significantly reduced serum TGF-β1, VEGF level and GBM thickness^27^. The correlation mechanisms of SWJH maybe also present in db/db mice. Therefore, the objective of the present study could explain the underlying protective effects and molecular regulation on diabetic renal injury by SWJH treatment in db/db mice. (Technical roadmap of this article, Figure 1).

## Materials and Methods

All methods described in this article were performed in accordance with the relevant guidelines and regulations.

### Materials

Curcumae Longae Rhizoma (No. 2017001, *Curcuma longa* L.), Phyllanthi Fructus (No. 2017001, *Phyllanthus emblica* L.), Tribuli Fructus (No. 2017001, *Tribulus terrestris* L.), these three herbals pieces purchased from the International Commercial Trade Market (a herbal medicine market, Chengdu, Sichuan, China) and carefully authenticated by professor (Xianrong Lai) of the School of Ethnic Medicine, Chengdu University of Traditional Chinese Medicine. Berberidis dictyophyllae Cortex (No. 2017001, *Berberis dictyophylla* F.) obtained from The Ganlu Traditional Tibetan Medicine Group Co., Ltd (Lasha, Tibet, China) and carefully authenticated using DNA barcode by associate professor (Gang Fan) and professor (XianRong Lai) of the School of Ethnic Medicine, Chengdu University of Traditional Chinese Medicine.

ELISA kit for serum TGF-β1 (No. 70-EK2812/2) and VEGF (No. 70-EK2832/2) purchased from MultiSciences Biotech Co., Ltd (Hangzhou, Zhejiang, China). UAE kit (No. 20170222), Scr kit (No. 20160902), UA kit (No. 20170619), BUN kit (No. 20170428) and UMAlb kit (No. 20170222), the above quantitative kits purchased from Nanjing Jiancheng Bioengineering Institute (Nanjing, Jiangsu, China). Antibodies specific for TGF-β1 (No. ab92486), VEGF (No. ab46154) and HIF-1α (No. ab2185), purchased from Abcam trading Co., Ltd (Cambridge, England, UK). RevertAid First Strand cDNA Synthesis Kit (No. #K1622) purchased from Invitrogen-ThermoFisher (Waltham, Massachusetts, USA), FastStart Universal SYBR Green Master(Rox) mix kit (No. 04913914001) purchased from Roche (Basel, Basel, Switzerland). PBS (No. G0020), Diaminobenzidine (DAB, No. K5007), BCA protein quantitative test kit (No. G5001), Tris Buffered Saline (No. G0001), Trizol reagent (No. G3013), 4% paraformaldehyde (No. 171820), glyceraldehyde-3-phosphate dehydrogenase (GAPDH, No. GB12002), β-actin (No. GB12001) purchased from Wuhan Servicebio technology Co., Ltd (Wuhan, Hubei, China).

### Extract preparation and quality control of SWJH extract

**Figure 2:**
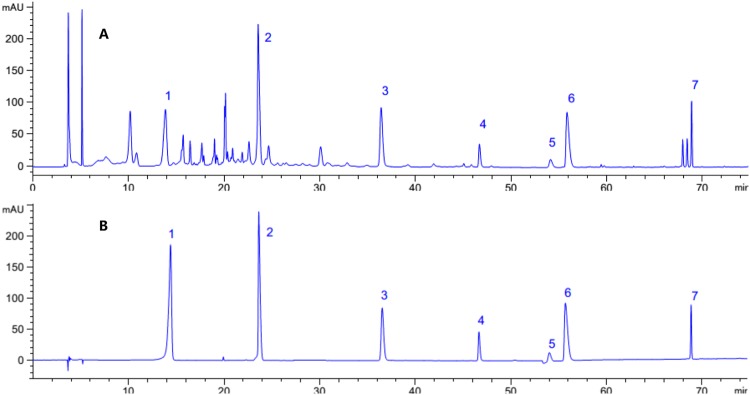
HPLC chromatogram for SWJH (**A**) and mixed reference substance (**B**). 1. gallic acid; 2. magnoflorine; 3. ellagic acid; 4. Jatrorrhizine hydrochloride; 5. palmatine hydrochloride; 6. berberine hydrochloride; 7. curcumin.

SWJH formulated in a proportion of drugs used 1:2:1:2 (Curcumae Longae Rhizoma: Berberidis dictyophyllae Cortex: Phyllanthi Fructus: Tribuli Fructus), with the extract preparation and quality control methods of these herbal components performed as previously described^27^. The high-performance liquid chromatography (HPLC) analysis method of SWJH performed as previously reported (Figure 2)^27^. The content of gallic acid, magnoflorine, ellagic acid, Jatrorrhizine hydrochloride, palmatine hydrochloride, berberine hydrochloride and curcumin in SWJH determined as 7.6482 mg/g, 13.8024 mg/g, 3.7290 mg/g, 0.9316 mg/g, 0.6789 mg/g, 4.6780 mg/g and 3.7890 mg/g, respectively.

### Animal experiment

All animal experiments performed with the approval of the Institutional Animal Care and Use Committee of Chengdu University of Traditional Chinese Medicine (Record number: 2015-03). All animal performed and purchased with the approval of National Resource Center of Model Mice and Model Animal Research Center of Nanjing University (Approval Number: SCXK (Su) 2015-0001, NRCMM&MARC, Nanjing, China). Eight week-old male db/db mice (BKS.Cg-Dock7^m+^/^+^Lepr^db^/JNju, type 2 diabetic mice model), their normal littermates (db/m, wild-type) purchased from NRCMM&MARC. All animals were adaptive breeding for two weeks in an air-conditioned animal experiment room according to China standard at (20–24 °C) and humidity level (60–70%) with a 12-hour light and dark cycle with free access to water and food (Animal observation room in School of Pharmacy, Chengdu University of Traditional Chinese Medicine, Chengdu, China, Approval Number: SYXK (Chuan) 2014-0124). All db/db mice with FBG levels above 16.7 mmol/L, experimental mice randomly allocated to the 6 groups (n = 6 per group): Diabetic control (db/db mice); low, middle and high dose of SWJH (0.978, 1.957 and 3.914 g/kg, respectively); berberine group; and positive control (0.196 g/kg metformin). The same age-matched db/m mice used as normal control (db/m). Diabetic and normal control groups treated with the identical volume normal physiological saline, all treatment groups administered treated by SWJH with intra-gastric gavage (0.1 ml/10 g) once a day. Amount of food and water intakes recorded, fasting blood glucose (FBG) measured using a blood glucose meter (Roche, Switzerland) in the tail vein blood once 2 weeks, for 8 weeks. The kidneys removed, weighed and washed with PBS, the right kidney dissected and frozen at −80 °C until processed for western blot and real-time PCR analyses. The left kidney fixed with 4% paraformaldehyde for histological examination, periodic acid-silver methenamine (PASM) and immunohistochemistry staining.

### Biochemical analysis

After 8 weeks treated with SWJH, all animals kept in the metabolic cages (Suzhou, China) for 24-hour urine collection and detected FBG levels prior to the sacrifice. Blood samples collected via cardiac puncture and then centrifuged at 3,000 revolutions per minute (rpm) for 15 min to obtain serum. The serum was stored at −80 °C until use. Urine albumin excretion (UAE), serum creatinine (Scr), uric acid (UA), blood urea nitrogen (BUN) and urine microalbumin (UMAlb) measured by corresponding quantitative kit, respectively. Serum TGF-β1 and VEGF measured by corresponding ELISA kit.

### Histological examination

The left kidneys used to evaluate renal lesions. Renal tissues fixed in 4% paraformaldehyde and embedded in paraffin, hematoxylin and eosin (H&E) performed at 200× magnification and PASM stained at 400× magnification, both on paraffin sections(5-μm thickness) by an observer blinded from which the tissue slices originated. Mean IOD of GBM thickness in PASM-stained sections determined using the Image-Pro Plus 6.0 (Media Cybernetics) quantitative software from DM1000 (Leica, Germany)^28,29^.

### Immunohistochemistry analysis

After antigen retrieval and blocking, paraffin-embedded kidney sections (5-μm thickness) incubated overnight at 4 °C with primary antibodies for TGF-β1, VEGF and HIF-1α(diluted 1,000 times with PBS) as previously described^30,31^, and then washed with PBS and incubated for 50 min with secondary antibodies (diluted 3,000 times with PBS), then used diaminobenzidine (DAB) coloration. The immunostaining signals within each selected glomerular or tubular-interstitialarea were highlighted and quantified using Image-ProPlus 6.0 quantitative software as described above.

### Real-time PCR analysis

Total RNA extracted from kidneys, respectively, cDNA synthesized using RevertAid First Strand cDNA Synthesis Kit. Quantitative real-time PCR performed using the FastStart Universal SYBR Green Master mix kit with the primers listed in Table 1 on the StepOnePlus Real-Time PCR System (Applied Biosystems&Thermo Fisher Scientific, Canada). As mentioned above to use 2^−ΔΔCt^ method to calculate relative mRNA expression level^32,33^. The expression of GAPDH mRNA was used as internal reference control.

**Table 1:** Primers list for real-time PCR analysis.

Gene	Forward 5′-3′	Reverse 5′-3′	Product length
HIF-1α	TTGCTTTGATGTGGATAGCGATA	CATACTTGGAGGGCTTGGAGAAT	223
TGF-β1	CGAAGCGGACTACTATGCTAAAGAG	TGGTTTTCTCATAGATGGCGTTG	77
VEGF	GTAACGATGAAGCCCTGGAGTG	CACAGTGAACGCTCCAGGATTTA	243
GAPDH	AGGAGCGAGACCCCACTAACA	AGGGGGGCTAAGCAGTTGGT	247

### Western blot analysis

The kidneys dissociated using 10% PBS buffer solution, total protein concentration measured by BCA protein quantitative test kit. Proteins in the lysates separated on a SDS-PAGE gel and electro-blotted proteins onto nitrocellulose membranes as previously described^32–34^. After 1-hour blocking in a 5% non-fat milk, membranes incubated overnight at 4 °C with primary antibodies (diluted 1,000 times with TBST, a TBS with 0.1% Tween-20). Blots washed with TBS and incubated at room temperature for 1-hour with secondary antibodies (diluted 3,000 times with TBST). After washed, protein bands detected using the alphaEaseFC(Alpha Innotech, USA) and the PhotoShop(Adobe, USA), β-actin used as a loading control. Densitometry analysis performed and expressed as the integrated optical density relative to β-actin.

### Statistics

All data expressed as the mean ± SD. Statistical analyses conducted using SPSS version 22.0 for Windows (IBM, USA) to compare between the groups by one-way analysis of variance (ANOVA). A value of *P* < 0.05 was considered statistically significant. All data transformed by napierian logarithm standardize method ( = LN(x)/LN(MAX)), principal component analysis (PCA) and partial least squares regression (PLS) and hierarchical cluster analysis (HCA) used the Unscrambler X (CAMO, Oslo, Norway).

## Results

### Results for biological chemistry

Compared with the db/m group, bodyweight and left kidney weight significantly higher in the db/db group, while middle, high dosage groups treated with SWJH and Berberine group significantly decreased bodyweight, and all treatment groups significantly decreased kidney weight (Table 2).

**Table 2:** Biochemical data of all groups.

Groups	db/m	db/db	Metformin (0.196 g/kg)	SWJH (0.978 g/kg)	SWJH (1.957 g/kg)	SWJH (3.914 g/kg)	Berberine (0.157 g/kg)
Body weight (g)	25.2 ± 1.00**	60.7 ± 3.50	58.9 ± 5.70	57.6 ± 2.43	53.3 ± 2.10**	56.0 ± 2.80*	56.5 ± 3.00*
Left kidney weight (g)	0.17 ± 0.01**	0.22 ± 0.01	0.18 ± 0.02**	0.18 ± 0.03*	0.18 ± 0.01**	0.18 ± 0.02**	0.18 ± 0.01**
FBG (mmol/L)	6.0 ± 0.60**	26.5 ± 1.90	17.9 ± 3.10**	23.9 ± 4.00	18.9 ± 5.80*	17.2 ± 7.90*	17 ± 2.10**
BUN (mmol/L)	4.6 ± 0.40**	7.4 ± 1.1	6.1 ± 0.7*	5.1 ± 0.4**	4.6 ± 1.1**	5.0 ± 0.7**	4.7 ± 0.7**
UAE (mg/day)	0.64 ± 0.04**	5.04 ± 1.26	1.40 ± 0.73**	1.69 ± 0.67**	1.14 ± 0.45**	0.78 ± 0.12**	0.60 ± 0.33**
UMAlb (µg/day)	9.5 ± 1.7**	63.6 ± 25.4	28.6 ± 16.7*	20.9 ± 9.3**	33.1 ± 20.4*	18.8 ± 9.8**	16.9 ± 3.8**
Scr (µmol/L)	63.0 ± 5.9**	147.8 ± 14.2	119.1 ± 5.0**	124.4 ± 20.0*	115.2 ± 7.6**	118.9 ± 17.3*	64.3 ± 14.8**
UA(µmol/L)	66.3 ± 13.1**	152.6 ± 41.1	75.9 ± 14.7**	73.7 ± 31.6**	101.2 ± 24.7*	109.3 ± 43.9	96.6 ± 15.7*
TGF-β1 (×1000 pg/ml)	6.2 ± 3.0**	16.6 ± 5.8	6.5 ± 3.0**	4.7 ± 0.9**	5.4 ± 0.8**	7.8 ± 3.0*	5.3 ± 1.1**
VEGF (pg/ml)	184.5 ± 17.2*	205.7 ± 5.9	212.0 ± 22.8	179.2 ± 28.8	224.7 ± 25.7	197.1 ± 34.1	188.1 ± 17.3*

Data expressed as mean ± SD, *P < 0.05, **P < 0.01 compared with db/db group (n = 6).

The levels of FBG, BUN, also significantly increased in db/db mice group compared to the corresponding values in db/m mice group (Table 2), the middle, high dose groups treated with SWJH, and Berberine group significantly reduced the levels of FBG, at the same time, all treatment groups significantly down-regulated the levels of BUN (Table 2).

In addition, the levels of the UAE, UMAlb, Scr, UA, TGF-β1 and VEGF were also significantly increased in the db/db group compared to the db/m group, all groups with SWJH treatment, and Berberine group significantly lowered the levels of the UAE, UMAlb, Scr and TGF-β1, while Berberine group significantly decreased the levels of Serum VEGF and UA, low and middle groups of SWJH treatment significantly decreased the levels of UA (Table 2).

### Results for histopathology

**Figure 3:**
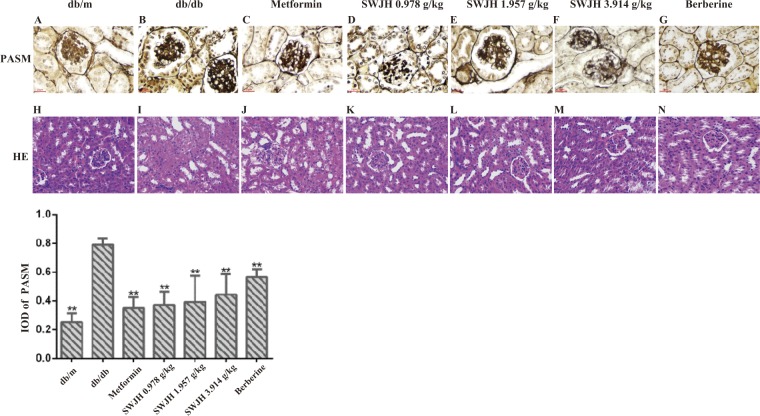
Effect of SWJH on renal histopathology and ultrastructural pathology. (**H**–**N**) H&E stain at 200× magnification. (**A**–**G**) PASM staining at 400× magnification. (**O**) Mean IOD of GBM in PASM staining. **P < 0.01 compared with db/db group (n = 6).

H&E staining result demonstrated that compared to the db/m mice group, the db/db mice group had notable renal tubule epithelial cells edema, renal capsule stenosis, epithelial cells shedding, and fatty degeneration (Figure 3). All treatment groups with SWJH and berberine significantly ameliorated those changes. Additionally, PASM staining demonstrated that compared to the db/m mice group, the db/db mice group had notable increasing in mean IOD, which shown that GBM thickness were higher in db/db mice group than in db/m mice group (Figure 3), whereas all treatment groups with SWJH significantly lowered the GBM thickness in db/db mice (Figure 3).

### Results for immunohistochemistry

**Figure 4:**
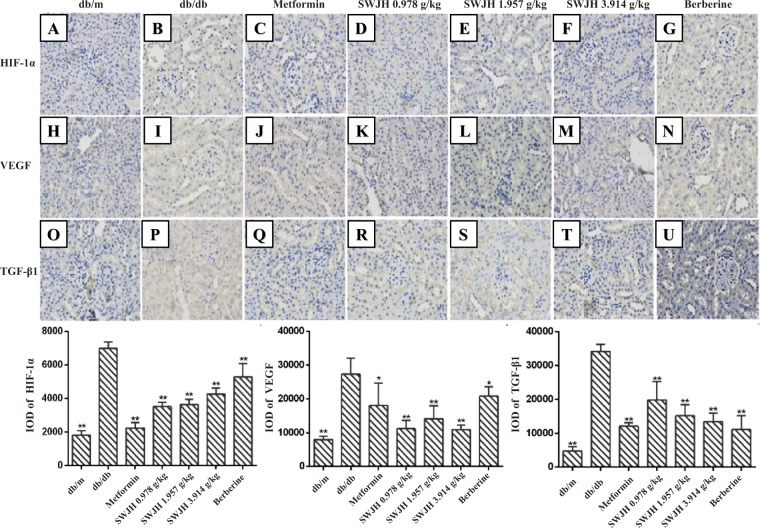
Effect of SWJH on renal HIF-1α/VEGF/TGF-β1 expression. (**A**–**U**) Immunohistochemistry of HIF-1α, VEGF and TGF-β1. Original magnification (**A**–**U**) at 400× magnification; (**A**–**C**) Quantitative analyses of immunohistochemical staining of HIF-1α, VEGF and TGF-β1. *P < 0.05, **P < 0.01 compared with db/db group (n = 6).

Results revealed the Integral optical density value (IOD) increased in db/db mice group, which shown the protein level of HIF-1α, VEGF and TGF-β1 upregulated in the kidney of db/db mice (Figure 4). These changes, however, significantly alleviated in all treatment groups, compared with the control db/db mice group (Figure 4).

### Results for real-time PCR

**Figure 5:**
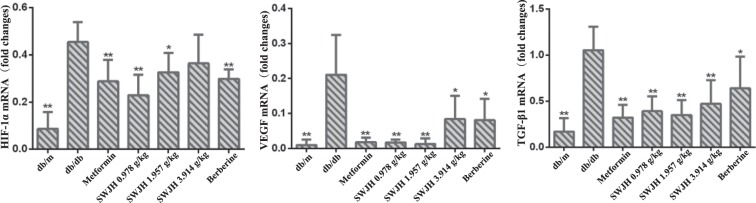
Effect of SWJH on renal HIF-1α, VEGF and TGF-β1 expression. Real-time PCR analyses of HIF-1α, VEGF and TGF-β1 mRNA levels. *P < 0.05, **P < 0.01 compared with db/db group (n = 6).

HIF-1α, TGF-β1, and VEGF have been identified as the key regulators of renal fibrosis in DN. To elucidate the mechanism of SWJH inhibiting renal fibrosis, we examined the effects of SWJH on the expression of HIF-1α, TGF-β1, and VEGF in diabetic kidneys using real-time PCR. As data shown (Figure 4), compared to the db/m mice group, the db/db mice group represented increased renal expression of HIF-1α, TGF-β1, and VEGF mRNA. After 8 weeks, low, middle doses groups treated with SWJH and Berberine group significantly decreased renal expression of HIF-1α, all treatment groups significantly decreased renal expression of TGF-β1, and VEGF mRNA in db/db mice (Figure 5).

### Results for western blot

**Figure 6:**
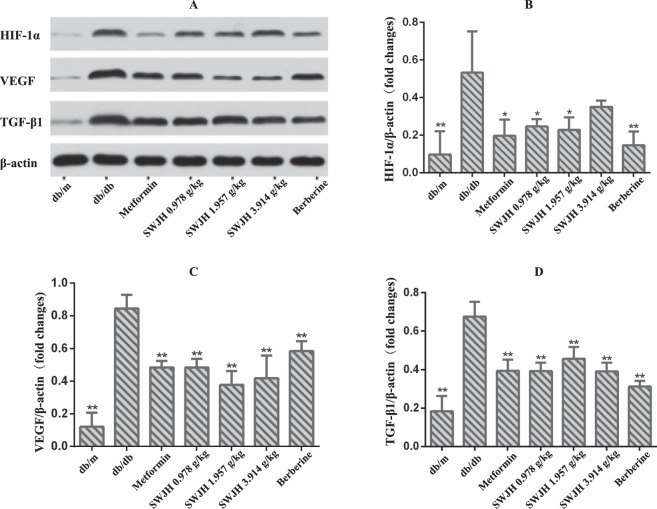
Effect of SWJH on renal HIF-1α, VEGF and TGF-β1 expression. (**A**–**D**) Western blot analyses of HIF-1α, VEGF and TGF-β1 protein levels. **P < 0.01 compared with db/db group (n = 6).

Since vascular endothelial injury is the common pathogenesis of DN. However, HIF-1α, TGF-β1, and VEGF are important components of DN angiogenesis. Examined with western blots, as data shown (Figure 5), expression of HIF-1α, TGF-β1, and VEGF proteins significantly increased in db/db mice group. All groups treated with SWJH and Berberine group significantly decreased renal expression of TGF-β1, and VEGF in db/db mice, while Berberine group, low and middle groups of SWJH treatment significantly decreased the levels of HIF-1α (Figure 6).

### Statistical results with PCA, PLS and HCA

**Figure 7:**
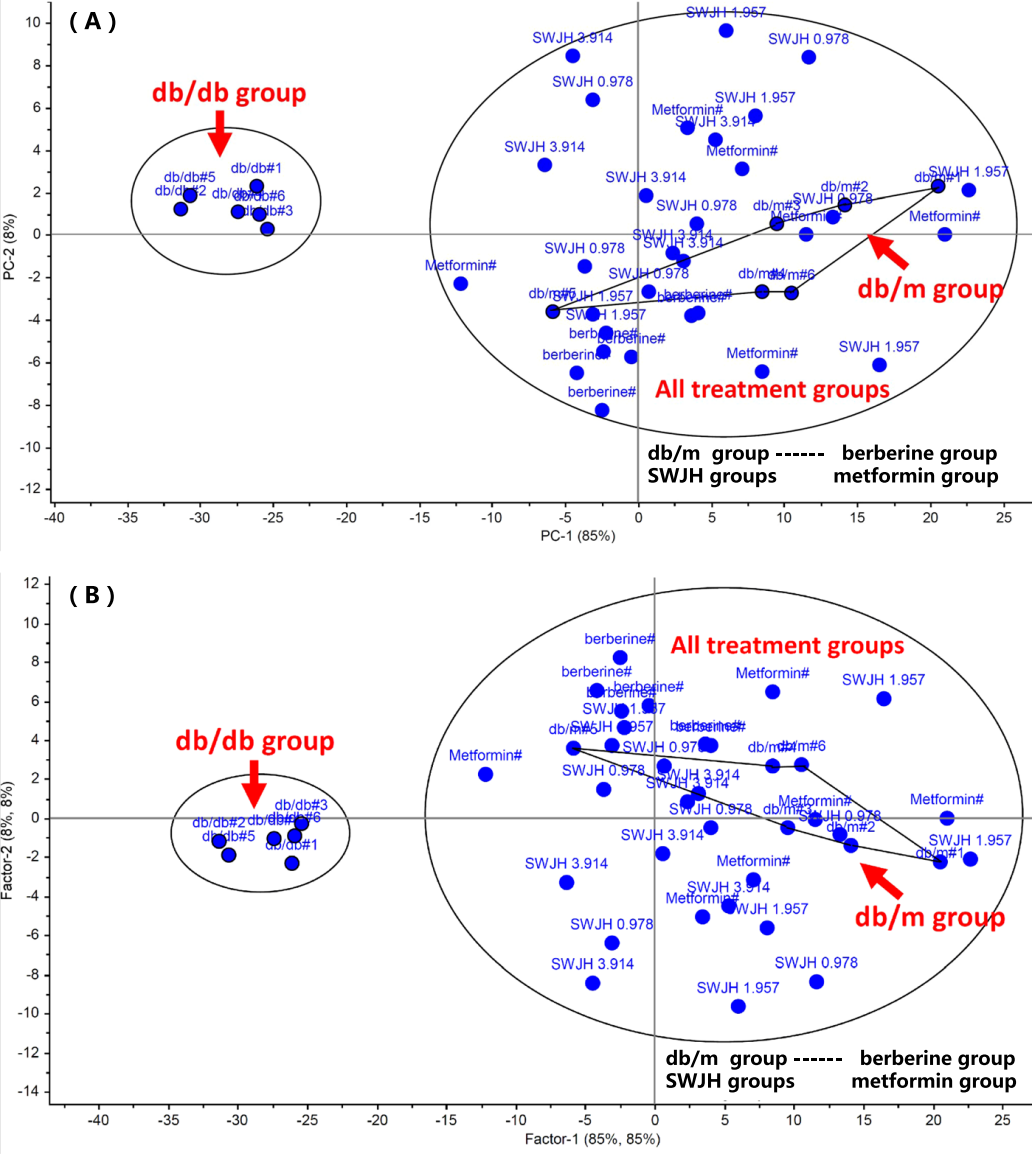
PCA (**A**) and PLS (**B**) for effect of SWJH.

**Figure 8:**
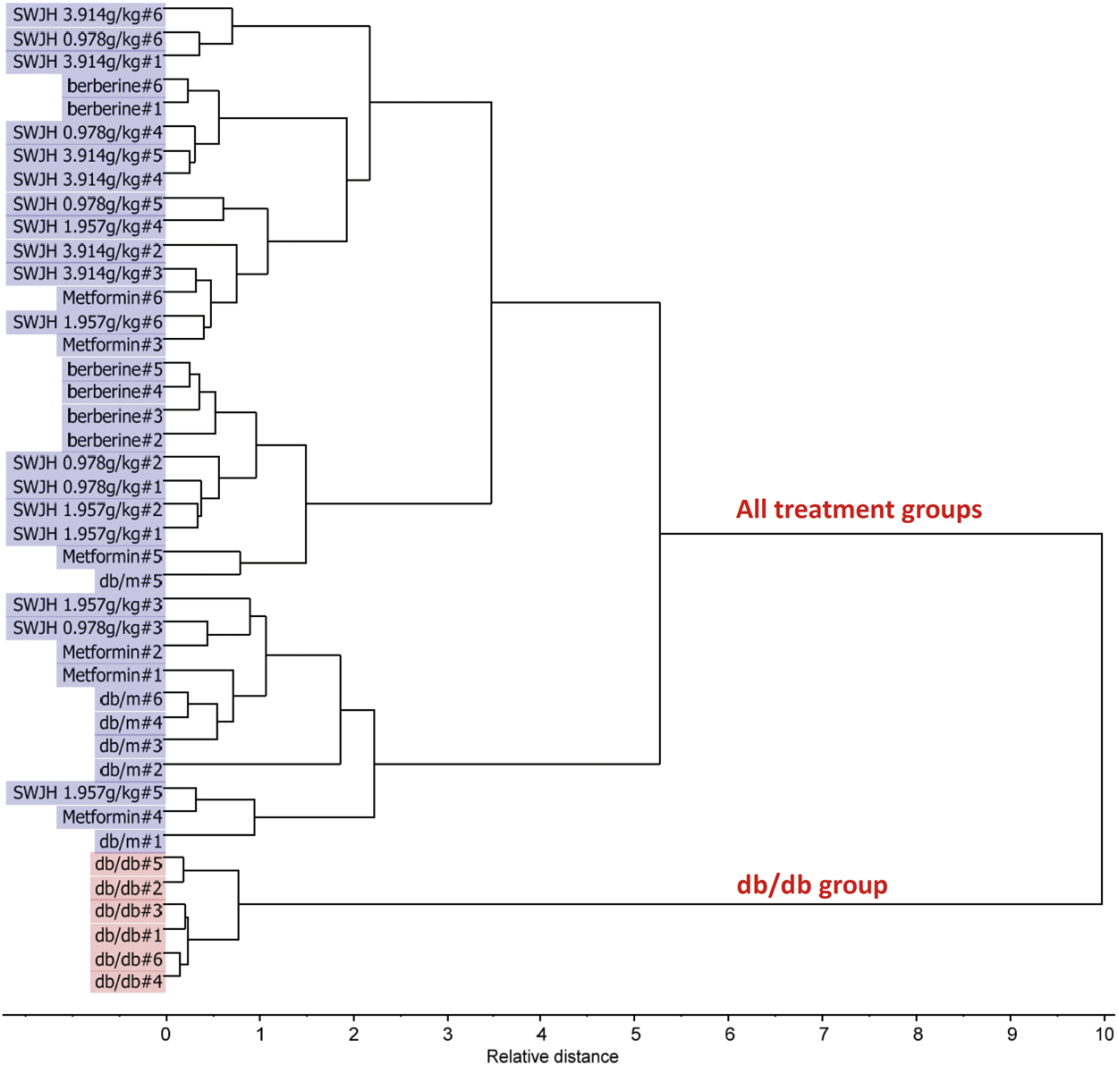
HCA by Dendrogram plot, Squared Euclidean and Ward’s method for effect of SWJH.

PCA, PLS, HCA provided with The Unscrambler X, are unsupervised multivariate data analysis methods and gives the comprehensive view of the clustering trend for the multidimensional data^35–39^, the results shown that all treatment groups, significantly ameliorated (separated, divided) from db/db group as two different class group, shown that the pharmacodynamic parameters of db/db mice significantly improved to varying degrees. Besides, all treatment groups clustered into a class of blank group, db/m, shown that SWJH, metformin, berberine maybe play the same role on DN, SWJH was a effective drug for DN, therefore it is necessary to carry out an in-depth study (Figures 7, 8).

## Discussion

DN, a major microvascular complication of diabetes, which is responsible for morbidity and mortality in diabetic patients^40^. Strict control of FBG levels in diabetics fail to prevent the progression of kidney diseases, indicating that DN pathogenesis are both complex and unclear, despite the relative large number of studies^41^. Hence, it is a critical need to develop novel reagents to improve DN therapy.

In our studies, we demonstrated that 24-hour proteinuria degree and mean IOD of GBM obviously elevated in db/db mice. GBM thickness increased can cause GFB dysfunction and then contribute to the onset and development of micro-albuminuria^42–44^. The data for evidence-based medicine suggested that although intensive FBG control, proteinuria occurred inevitably and kept increasing at the early stage of DN^45,46^. However, SWJH treatment effectively reduced the amount of albuminuria and GBM thickness in db/db mice. What’s more, SWJH treatment normalized Scr, BUN, UA, and decreased FBG level in db/db mice. These results indicated that SWJH shown significant protection effects and against diabetic renal injury.

TGF-β1 has confirmed to play a central role in renal fibrosis^47–50^. Expression of TGF-β1 protein in the db/db mice remarkably upregulated, and SWJH treatment markedly inhibited the protein expression and mRNA abundance of TGF-β1 in db/db mice kidney. These results suggested that SWJH negatively regulates the TGF-β1 expression to control renal fibrosis in DN.

HIF-1α is a highly oxygen sensitive monitor of regulatory protein in the body. A large of evidence indicates that HIF-1α is a key cytokine of renal sclerosis in hyperglycemic conditions^51^. Our study found that HIF-1α mRNA and protein were over-expressed in db/db mice, suggesting that HIF-1α closely associated with DN. SWJH treatment remarkably inhibited the protein and mRNA expression of HIF-1a in db/db diabetic mice kidney.

Many studies have suggested that chronic angiogenesis and vascular endothelial dysfunction is very important in the occurrence and development of DN^13^. The correlation between VEGF level and DN is a popular research topic. VEGF increased the permeability of vascular endothelial cells and changed the GFB^52^. Our study confirmed that VEGF mRNA and protein expression were significantly enhanced in db/db mice, VEGF overexpression caused GFB damage seriously and elevated permeability, promoted TGF-β1, HIF-1α, Scr, BUN, and UMAlb secretion at the high level. It further induces renal hypoxia, resulting in 24-hour proteinuria and increased Scr and BUN secretion. VEGF can also aggravate renal tubular basement membrane thickness, glomerular sclerosis, and renal interstitial fibrosis by promote the extracellular matrix deposition, leading to renal function damage^53–55^. SWJH treatment normalized overexpression of HIF-1α and VEGF.

Compared with db/m mice, results of immunohistochemistry, Western Blot and RT-PCR shown that there was a large number of expression of HIF-1α, TGF-β1 and VEGF in db/db mice, may interact and promote each other in the development of DN. During the whole pathological process, hyperglycemic up-regulation of HIF-1α expression is the intermediate link in the occurrence of DN, which leading to the overexpression of VEGF and causing glomerular capillary dysfunction and leading to proteinuria, as well known, increase of UAER is an important sign of endothelial dysfunction and small vessel disease. Moreover, with the development of DN, TGF-β1 plays a central role in the pathogenesis of DN in the process of renal fibrosis, eventually leading to ESRD. Taken together, HIF-1α, VEGF and TGF-β1 played important roles in the occurrence and development of DN that contributed to ESRD.

These results suggested that SWJH, a mixture comprising multiple herbal compounds, maybe act on a number of target points simultaneously to generate a combined comprehensive effect, which may exhibit distinct advantages for the treatment of DN compared to agents specific for a single molecular target. As a multi-component herbal mixture, SWJH can be prepared using standardized methods and under strict quality control, thereby presenting a more appropriate therapeutic drug. However, these evidences shown the ability of SWJH to ameliorate renal fibrosis, obtained from studies in animal, needs to be confirmed with further research in human DN.

## Conclusion

In conclusion, our current study suggests that SWJH prevented hyperglycemia, renal fibrosis, and alleviated GBM thickness, thus ameliorating renal functional in db/db mice. The increased of TGF-β1, HIF-1a and VEGF maybe associate with diabetic renal damage in DN, and maybe play important roles interact with the occurrence and development of DN. The underlying mechanism was associated with DN by inhibiting the TGF-β1, VEGF and HIF-1α overexpression. However, DN is a complex disease, many of the targets involved, we just investigated only few of them on SWJH treatment in db/db mice, but we considered that SWJH ameliorated DN obviously in clinical treatment. Therefore, Tibetan formula SWJH, a multiple herbal compounds, may be a useful drug for DN treatment, which worthy to make further explored.
